# Extending the Reach of Tooling Theory: A Neurocognitive and Phylogenetic Perspective

**DOI:** 10.1111/tops.12554

**Published:** 2021-06-24

**Authors:** Jennifer A. D. Colbourne, Alice M. I. Auersperg, Megan L. Lambert, Ludwig Huber, Christoph J. Völter

**Affiliations:** Comparative Cognition Unit, Messerli Research Institute, University of Veterinary Medicine Vienna, University of Vienna, Medical University of Vienna

**Keywords:** Animal behavior, Animal tool use, Evolution of technology, Comparative cognition, Object manipulation, Spatial relations, Tooling, Replication crisis

## Abstract

Tool use research has suffered from a lack of consistent theoretical frameworks. There is a plethora of tool use definitions and the most widespread ones are so inclusive that the behaviors that fall under them arguably do not have much in common. The situation is aggravated by the prevalence of anecdotes, which have played an undue role in the literature. In order to provide a more rigorous foundation for research and to advance our understanding of the interrelation between tool use and cognition, we suggest the adoption of Fragaszy and Mangalam’s (2018) tooling framework, which is characterized by the creation of a body-plus-object system that manages a mechanical interface between tool and surface. Tooling is limited to a narrower suite of behaviors than tool use, which might facilitate its neurocognitive investigation. Indeed, evidence in the literature indicates that tooling has distinct neurocognitive underpinnings not shared by other activities typically classified as tool use, at least in primates. In order to understand the extent of tooling incidences in previous research, we systematically surveyed the comprehensive tool use catalog by Shumaker et al. (2011). We identified 201 tool use submodes, of which only 81 could be classified as tooling, and the majority of the tool use examples across species were poorly supported by evidence. Furthermore, tooling appears to be phylogenetically less widespread than tool use, with the greatest variability found in the primate order. However, in order to confirm these findings and to understand the evolution and neurocognitive mechanisms of tooling, more systematic research will be required in the future, particularly with currently underrepresented taxa.

## 1 Introduction

In order to achieve a better understanding of the evolution of technology, it is critical to study how tool use has emerged across different species. So far, comparative tool use research has largely focused on collecting and categorizing examples of tool use behaviors in different nonhuman animals (hereafter, animals). Emblematic of this approach is the book *Animal Tool Behavior* by Beck (1980), updated by Shumaker, Walkup, and Beck in 2011, which ambitiously strove to compile every reference to tool-related behavior in the animal literature. While the cases they identified were divided by 22 modes (e.g., “Throw,” “Pound,” “Reach,” etc.), the authors made an intentional choice not to focus on issues of cognition, evolution, or ontogeny, considering such topics beyond the scope of the book. With currently over 1,700 citations (source: Google Scholar) between the original and updated versions, *Animal Tool Behavior* has had an unrivalled impact on the field, and its definition of tool use has been considered the most influential (Crain, Giray, & Abramson, 2013; St. Amant & Horton, 2008). In the book, tool use is defined as:

The external employment of an unattached or manipulable attached environmental object to alter more efficiently the form, position, or condition of another object, another organism, or the user itself, when the user holds and directly manipulates the tool during or prior to use and is responsible for the proper and effective orientation of the tool. (Shumaker et al., 2011, p. 5)

Although this definition has been widely adopted, it has, like most tool use definitions, come under a fair amount of criticism; so much so, that Shumaker et al. (2011) updated it to take into account specific arguments put forth by various authors. For example, in Beck’s (1980) original version of the book, a true tool had to be “unattached,” but as St. Amant and Horton (2008) argued, a tool can be attached but still freely manipulable, such as a hook attached to a rope, so Shumaker et al. (2011) altered their definition to “the external employment of an unattached or *manipulable attached* environmental object” (p. 5). Debates over similar technicalities have been ongoing, as different authors have attempted to create a conclusive definition of tool use. Crain et al. (2013) have provided an excellent overview of the issue, noting that among the most popular definitions, not one single criterion of tool use was shared by all; each definition had its own distinct specifications for which object types, employment, and tasks could be said to constitute tool use. And so, the field appears to be moving in circles as it debates the small details of specific cases that are considered tool use by some definitions but not others, rather than working toward any kind of consensus. As a result, the lack of agreement and ambiguity around tool use has allowed the proliferation of questionable cases as researchers can select the definition that best fits the behavior they are describing, or, in a surprising number of cases, as discovered by Crain et al. (2013) and Shumaker et al. (2011), avoid providing an explicit definition at all.

This current state of overly broad and vague definitions has created ongoing problems, as more and more ambiguous cases have been published. In 2020, there has been a wide variety of animal “tool use” studies, including a study about honey bees (*Apis cerana*) that use feces as a tool to defend nest entrances (Mattila et al., 2020), domestic dogs (*Canis lupus familiaris*) that chew on sticks as a tool to maintain oral hygiene (Brooks & Yamamoto, 2021), and the placement of sand structures by black imported fire ants (*Solenopsis richteri*) as a tool to more safely acquire liquid food (Zhou, Du, & Chen, 2020). Although each of the authors contextualized their findings within preexisting tool use research, without a coherent underlying framework, it is impossible to interpret the significance of each behavior and how they might relate to each other, because they represent such distinct behavioral phenomena. This is not to say that these are not complex or cognitively interesting behaviors, but if nearly every action with an object can be argued to fulfill the criteria for the steadily broadening definitions of tool use, then we run the risk of camouflaging its real role in the evolution of cognition and technology.

In addition to conceptual ambiguity, the tool use literature is full of cases with very thin empirical support. This is evident in Shumaker et al. (2011), which catalogs all tool use reports, many of which are only based on a single study or even a single anecdote. Moreover, the amount of detailed research invested in studying tool use experimentally is biased toward primates. These issues can blur the picture and make it more difficult to identify potentially meaningful phylogenetic patterns of tool use throughout the animal kingdom. Even more concerning is that any reports of tool use events discovered in new species may be widely publicized, especially because such stories have a strong appeal to the media and its consumers, as humans have long considered tools to have had a special place in our history and evolutionary development. A recent publication on tool use in puffins (*Fratercula arctica*) exemplifies the impact of anecdotal evidence on the tool use literature, in which two observations of puffins apparently using a stick to scratch their bodies were reported (Fayet, Hansen, & Biro, 2019). The article describing these observations received substantial media attention, which repeated controversial suggestions made by the authors, such as that the puffins were intentionally using sticks to dislodge ticks, and that they may be comparable in their innovative and cognitive abilities to parrots and corvids despite having a smaller brain (Fayet et al., 2019). Moreover, they suggested that the findings should considerably impact our present understanding of the selection pressures and the role of cognition involved in the onset of avian tool behavior (Fayet et al., 2019). Yet these conclusions were the result of an anecdotal observation from a far distance in 2014, and an 11-s video clip, of which less than a second showed the stick making contact with the bird’s breast (Auersperg, Schwing, Mioduszewska, O’Hara, & Huber, 2020). It has been argued that the more parsimonious interpretation was that the stick incidentally made contact when the bird either tried to scratch itself with its beak (Auersperg et al., 2020; Farrar, 2020a) or shook its head (Sándor & Miklósi, 2020). Eliminating these possibilities requires more rigorous research and stronger evidence, particularly when such far-reaching conclusions are drawn.

While we fully acknowledge that anecdotes are vital to the formation of new hypotheses, and should absolutely be reported to stimulate research ideas, we believe that incorporating such findings into comparative discussions on the evolution of tool behavior without a fair amount of evidence can have seriously harmful ramifications. As the replication crisis is sweeping over scientific disciplines, it is becoming clear that in comparative cognition, evidentiary and publication standards also need to become more stringent (Beran, 2018; Farrar, Boeckle, & Clayton, 2020b; Stevens, 2017) and robust theoretical frameworks need to be developed that enable the deduction of clear predictions (Muthukrishna & Henrich, 2019). In tool use research, we argue that this can be best accomplished by (a) requiring stricter evidence for extraordinary claims, (b) refraining from making assumptions about a species in the absence of such evidence, and (c) advancing theoretically by developing definitions of object manipulation that are more coherent at a cognitive and neuroscientific level. As we will elaborate in the following section, Fragaszy and Mangalam’s (2018) tooling framework is a promising candidate in this last respect.

## 2 The tooling framework

Rather than continuing the current unproductive cycle of readjusting tool use definitions, which has resulted in increasing ambiguity and muddiness around tool use, Fragaszy and Mangalam (2018) have made a radical proposal to start fresh, and completely reconceptualize the phenomenon. Instead of the traditional approach of focusing on the object and its use, they propose that biomechanical and spatial criteria should be considered instead to determine when an object is being used as a tool. The focus thus shifts from the object to the action, which is epitomized by their creation of a verb to represent this action: “to tool.” Fragaszy and Mangalam (2018) define the act of “tooling” as follows:

Tooling is deliberately producing a mechanical effect upon a target object/surface by first grasping an object, thus transforming the body into the body-plus-object system, and then using the body-plus-object system to manage (at least one) spatial relation(s) between a grasped object and a target object/surface, creating a mechanical interface between the two. (p. 194)

There are several important features of this definition that set it apart from traditional approaches. One is the necessity for the “creation of a mechanical interface” between the tool and the target while the tool is being held by the actor. While similar to St. Amant and Horton’s (2008) requirement for a “dynamic mechanical interaction” (p. 1203), what makes Fragaszy and Mangalam’s (2018) mechanical element novel is that there must be a managed interface created between the tool and the target, which necessitates that the tool is held by an engaged actor, whereas an interaction can take place at a distance. Immediately, the extent to which “tooling” is a radical reform becomes apparent, as several conventional modes of tool use are excluded with this qualification, particularly aimed throwing and dropping of objects. Thus, Aesop’s fable paradigm, in which the subject must drop stones to raise the water level in order to access a reward, does not qualify as tooling (e.g., Bird & Emery, 2009b; Cheke, Bird, & Clayton, 2011; Hanus, Mendes, Tennie, & Call, 2011; Jelbert, Taylor, Cheke, Clayton, & Gray, 2014; Mendes, Hanus, & Call, 2007; Stanton, Davis, Johnson, Gilbert, & Benson-Amram, 2017), nor do tasks requiring stone dropping to collapse a platform (e.g., Bird & Emery, 2009a; Ebel & Call, 2018; Laumer, Bugnyar, & Auersperg, 2016; Neilands, Jelbert, Breen, Schiestl, & Taylor, 2016; von Bayern, Heathcote, Rutz, & Kacelnik, 2009) or the aimed throwing of objects (e.g., Evans & Westergaard, 2004; Tokida, Tanaka, Takefushi, & Hagiwara, 1994; Westergaard & Suomi, 1994; Westergaard, Liv, Haynie, & Suomi, 2000).

However, it is important to note that whether these behaviors are considered tooling is not necessarily a reflection of their cognitive complexity, and so they remain interesting in their own right as unique, and possibly very sophisticated, forms of instrumental problem-solving and/or tool use outside the tooling framework. The most important example in this respect is perhaps aimed throwing. It is one of the most advanced and neurocognitively demanding forms of object interaction, requiring rapid muscle movements, trajectory calculations, and precise timing of release within a narrow launch window (Calvin, 1983). Unlike tooling, there is no ongoing feedback to allow for adjustments, thereby necessitating planning; once the object is thrown, the actor has no further control over the action outcome (Venkadesan & Mahadevan, 2017). Aimed throwing therefore involves unique challenges at the cognitive level, particularly concerning the planning, timing, and precision of the action. Although we share some basic throwing techniques with other primates (Westergaard et al., 2000), humans are unmatched among primates in their ability to throw at a high enough velocity to kill prey or conspecifics (Lombardo & Deaner, 2018). It has even been suggested that throwing specifically has played a unique role in the evolution of *Homo* (Bingham, 1999; Calvin, 1983, 1993; Roach, Venkadesan, Rainbow, & Lieberman, 2013; Schoenemann, 2006; Wood, Glynn, & Hauser, 2007). In any event, it is clear that aimed throwing is a qualitatively different tool use phenomenon than tooling, and should be considered separately and investigated on its own merits.

Thus, what truly sets tooling apart is not necessarily complexity, but the cognitive effects of “transforming the body into the body-plus-object system” (Fragaszy & Mangalam, 2018, p. 194). The body is a movement system (Higgins, 1977) with a limited set of possible motions (degrees of freedom) due to the physiological constraints of the joints (Bernstein, 1967). For example, the human elbow is greatly limited in its possible movements (flexion-extension and rotation) compared to an octopus tentacle, which has nearly infinite degrees of freedom. And so, grasping and engaging in the use of an object in order to tool not only requires coordination of the body’s degrees of freedom but also creates and redistributes the degrees of freedom as the possibilities for movement change (Fragaszy & Mangalam, 2018; Mangalam & Fragaszy, 2016). The locus of control for the action also shifts, from the appendage grasping the object to the part of the tool that makes contact while tooling (the end-effector), which, unlike a body part, offers no direct proprioceptive feedback (Arbib, Bonaiuto, Jacobs, & Frey, 2009; Mangalam & Fragaszy, 2016; Valk, Mouton, & Bongers, 2016). Thus, engaging in tooling transforms the movement system of the body in a distinctive manner that is absent in other forms of object use. Using a stick to probe into a hole changes the animal’s perceptuomotor interactions with the environment in a way that dropping a stone does not, even in cases in which the task demands of the latter are more difficult than the former.

The final integral piece of Fragaszy and Mangalam’s (2018) definition determines the complexity of tooling, that is “using the body-plus-object system to manage (at least one) spatial relation(s)” (p. 194). They hypothesize that tooling increases in difficulty according to (a) the number of spatial relations, (b) temporal order (with concurrent relations more difficult than sequential), (c) temporal dynamics (with dynamic relations more difficult than static), (d) frame of reference (with object/surface directed (allocentric) tooling more difficult than self-directed (egocentric) tooling), and (e) specificity (according to placement, orientation, and geometric alignment) (Fragaszy & Cummins-Sebree, 2005; Fragaszy & Mangalam, 2018). Measuring these aspects of tooling not only allows for a more detailed and coherent characterization of specific tool use phenomena but provides measures that can be applied across different species and modes of tool use for broader comparative purposes.

The added complexity of tooling has been illustrated by recent experimental work with great apes (bonobos (*Pan paniscus*), chimpanzees (*Pan troglodytes*), gorillas (*Gorilla gorilla*), and orangutans (*Pongo abelii*)): two studies compared how apes performed on trap tasks that either allowed them to navigate a food reward directly with their fingers through a vertical maze or required them to use a stick tool for moving the reward (Seed, Call, Emery, & Clayton, 2009; Völter & Call, 2014). The results suggested that tooling imposed a cognitive load on the apes: out of the individuals that did not have previous experience with the task (or a similar task), only a few of the tooling apes learned to avoid the traps, in contrast to the apes who could move the reward directly with their fingers. The tooling requirement specifically affected the apes’ performance in the acquisition phase, suggesting that the added complexity of tooling interferes with causal learning of novel problems. Seed et al. (2009) suggested that the added complexity resulted from the need to split attention (between the stick, reward, and the traps), cross-modal matching (e.g., missing tactile information on the continuity and solidity of the involved surfaces), and additional response variability.

Finally, tooling is required to be produced “deliberately” to achieve some goal. To do so might require the recognition of the tool’s affordances for acting in the environment. This may be facilitated, for example, in cases where a rake is prearranged with an object already placed directly inside of it (a classic Piagetian task, see Uzgiris & Hunt, 1975). However, this example would not constitute a case of tooling because the actor did not generate a mechanical interface between the tool and the target, but merely exploited a preexisting relation (Fragaszy & Cummins-Sebree, 2005). This highlights the importance of repeated observations and/or experimentation to establish a consistent ability to engage in tooling within a species; the difference between accidental use of an object versus goal-directed engagement in tooling cannot be distinguished by a single anecdote.

Along these lines, we believe that it is also useful to distinguish between flexible and inflexible stereotyped toolers, although this is not a part of the current tooling framework. While some species appear to be capable of using multiple tools in varied and novel contexts through learning and/or reasoning, others only engage in one mode in precise circumstances, increasing the likelihood that it is an evolved rather than innovative behavior (Call, 2013). It is the latter which Call (2013) considers comprising intelligence, as it requires “the ability to adapt to new situations with innovative solutions” (p. 17). He posits that flexible tool users possess three distinctive attributes that contribute to their creativity: (a) they seek out information through exploration, play, and latent learning; (b) they have a propensity for exploratively manipulating objects; and (c) they can restructure information gained from independent experiences, leading to insightful problem-solving (Call, 2013). This distinguishes these species from those for which tool use is a hardwired behavioral specialization. It is not an easy task to assess the degree of tooling flexibility across different species, further supporting the importance of conducting thorough experimental and observational studies before concluding the extent of a species’ abilities and their cognitive significance.

## 3 Extending tooling theory

The theory of tooling derives its key concepts from ecological psychology (e.g., affordances—Gibson, 1979) and movement science (e.g., degrees of freedom—Bernstein, 1967), and is considered to be “embodied” in that “no mediating representational processes are specified” (Fragaszy & Mangalam, 2020, p. 462); both problem and solution to a task are described according to the constraints of the individual, the task, and the environment (Fragaszy & Mangalam, 2020). Nevertheless, one of the main advantages of the tooling frame-work, and one that Fragaszy and Mangalam (2018) explicitly endorsed, is that it can be better integrated with other fields in ways that traditional tool use has not been able to, a task to which we will contribute by introducing a neurocognitive perspective.

We begin by separating the act of tooling into three distinct processes, according to the divisions set forth by Arbib et al. (2009): grasping, object manipulation, and the actual employment of the object for tooling. Grasping involves first recognizing the affordances that an object offers for grasping, moving the grasping appendage toward the object, and preshaping the appendage according to these affordances, and finally enclosing the object at the appropriate areas to establish the grasp (Arbib et al., 2009). Research with monkeys has revealed that this translation of the object’s surface affordances to a corresponding grip takes place in the AIP-F5 circuit, consisting of the anterior intraparietal (AIP) area of the posterior bank of the intraparietal sulcus and the closely connected F5 area of the premotor cortex (Castiello, 2005; Fagg & Arbib, 1998; Jeannerod, Arbib, Rizzolatti, & Sakata, 1995; Sakata, Taira, Kusunoki, Murata, & Tanaka, 1997); inactivation of either the AIP or F5 results in preshaping and grasping errors (Fogassi et al., 2001; Gallese, Murata, Kaseda, Niki, & Sakata, 1994). According to the Fagg–Arbib–Rizzolatti–Sakata (FARS) model, the AIP extracts an object’s affordances for grasping from the visual dorsal stream, while F5 selects the appropriate grasp. Which grasp is chosen may depend on how the object is intended to be used (Frey & Povinelli, 2012; Zander & Judge, 2015). This, in turn, is determined by higher order processing in the pre-frontal cortex (Arbib et al., 2009; Fagg & Arbib, 1998; Gerbella, Rozzi, & Rizzolatti, 2017). Although the majority of this research has been focused on hands, there is a class of F5 neurons that is active when either the hand or the mouth is used for grasping, indicating that the goal itself has been coded, not only the specific appendage engaged in the grasping (Arbib et al., 2009; Rizzolatti et al., 1988).

Manipulation of an object requires that, once it is grasped, the state of the object is changed in some way, such as its orientation, location, or shape (Arbib et al., 2009). There has been some research indicating a tendency for coordinated playful object combination and tool use in birds (Auersperg et al., 2015; Auersperg, Oswald, Domanegg, Gajdon, & Bugnyar, 2014; Gajdon, Lichtnegger, & Huber, 2014) and primates (Torigoe, 1985, 1987). One suggestion is that engaging in object manipulation enables learning about the functionality of various objects that can later be applied to problem-solving and tooling (Auersperg et al., 2015; Call, 2013; Gajdon, Ortner, Wolf, & Huber, 2013; Manrique, Gross, & Call, 2010, 2011; Visalberghi et al., 2009). A recent study by Lambert et al. (2017) on kea (*Nestor notabilis*) and New Caledonian crows (*Corvus moneduloides*) offers some support for this hypothesis, as subjects performed better on problem-solving tasks when they had an opportunity to explore the materials beforehand, compared to when they did not. Similarly, chimpanzees were found to be successful with a raking task only after having had prior experience manipulating sticks (Birch, 1945).

Object manipulation, however, seems to be distinct from tooling in the primate brain. Apraxia studies in humans indicate that conceptual knowledge of how a tool is used is separate from the acquired skills required to manipulate it (Johnson & Grafton, 2003; Johnson-Frey, 2004). Johnson and Grafton (2003) thus posit the existence of two independent systems in the parietal cortex, the “acting on” system for dexterous actions such as manipulating objects, and the “acting with” system, which contains tool use schemata. There is evidence that this “acting with” system involves the inferior frontal gyrus of the prefrontal cortex, an area which is also associated with categorical knowledge about tools (Chao & Martin, 2000; Grafton, Fadiga, Arbib, & Rizzolatti, 1997; Johnson & Grafton, 2003; Martin, Wiggs, Ungerleider, & Haxby, 1996). “Acting with” a tool, as opposed to “acting on” a nontooling object, seems to quite literally transform the brain into a body-plus-object system. In studies with macaques, it has been found that the visual receptive fields of the intraparietal sulcus’ neurons expand during tool use, encompassing the peripersonal space occupied by the tool “as if the image of the tool was incorporated into that of the hand” (Iriki, Tanaka, & Iwamura, 1996, p. 2326). Based on this discovery, the researchers postulated that during tool use there is an extension of the body schema (Iriki et al., 1996; Iriki, Tanaka, Obayashi, & Iwamura, 2001), conceived by Head & Holmes (1911) as the representation of the body and its parts. Notably, this extension only lasts shortly after the tool stops being useful, despite still being held in the hand (Iriki et al., 1996; Maravita & Iriki, 2004), suggesting that the intentional active use of an object as a tool is a significant factor in creating the body-plus-object system. Similarly, a study of neurons in the macaque F5 grasping motor system revealed that, once the use of the tool had been learned, during employment it became coded into the motor system as though it were an artificial hand, even if the actual hand was making opposite movements (Umiltà et al., 2008). Studies in humans have reinforced these findings (for a review, see Maravita & Iriki, 2004) and, recently, have provided compelling evidence that a sense of agency might be important for inducing changes in the body schema (D’Angelo, di Pellegrino, Seriani, Gallina, & Frassinetti, 2018). Tool-derived body schema plasticity is now a widely accepted phenomenon in the literature (Cardinali et al., 2009). It thus appears that van Lawick-Goodall’s (1971) early description of a tool as an extension of the body^1^ is quite literally accurate from a neurocognitive perspective, at least in primates.

As the tool acts as an extension of the body, it also changes the functional and motoric possibilities of the body as the degrees of freedom become redistributed, and the end-effector shifts from the grasping appendage to the tool (Fragaszy & Mangalam, 2018). Arbib et al. (2009) refer to the latter as “distalization,” as the locus of control moves away from the body to the tool. This distalization requires a shift in attention to a displaced visual target, from the appendage and its interface with the tool, to the tool and its interface with the tooling target, while still retaining motor control of the grasping appendage (Arbib et al., 2009). There is not necessarily a direct spatiomotor correspondence between the action of the grasping appendage and the tool, such as when the hand is using a power grip while the tool is in a precision grip (Arbib et al., 2009). This was nicely illustrated in a study by Umiltà et al. (2008) with southern pig-tailed macaques (*Macaca nemestrina*), in which subjects were trained to use pliers, which require the hand to squeeze closed to close the pliers, as well as reverse pliers, which require the hand to open in order to close the pliers. In other words, the hand is used in completely opposite ways to accomplish the same goal with the two tools. Using single-unit recordings, Umiltà et al. (2008) found two distinct categories of neurons in the primary motor cortex area F1: those that responded to the distal tooling goal of closing the normal and reverse pliers, and those that responded to the actual movements of the hand. Whether such phenomena also hold true in non-primate species, especially in some of the flexible tooling parrot species, such as kea (Auersperg, Huber, & Gajdon, 2011) and Goffin’s cockatoos (*Cacatua goffiniana*) (Auersperg, Szabo, von Bayern, & Kacelnik, 2012), would be especially interesting, but remains to be seen.

In sum, the key elements of Fragaszy and Mangalam’s (2018) conception of tooling appear to be supported by some of the major findings in the neuroscientific study of tool use, particularly the transformation of the body into the body-plus-object system, at least in primates. This transformative body-plus-object system is absent in several modes of instrumental object manipulation that have been previously considered tool use, such as throwing or propping- and-climbing, but are not considered tooling. This does not necessarily diminish the value or interest of these types of problem-solving, but it is clear that they are qualitatively different from tooling on many levels, including the neurocognitive, biomechanical, and behavioral. Importantly, such specific neurocognitive adaptations may also suggest that the capacity for tooling could have potentially impacted the evolution of technology differently from these other types of object-related behaviors.

## 4 Applying tooling theory: Analysis of *Animal Tool Behavior*

Without a doubt, Shumaker et al.’s (2011) catalog has served as a foundational text for comparative tool use research. As previously discussed, their definition has been widely adopted and their work heavily cited. Beginning with Beck (1980), Shumaker et al. (2011) compiled nearly a thousand cases of tool use from approximately 1750 sources, making the book an incredibly comprehensive and invaluable resource to the tool use community. As the goal of this work was to collect and organize all preexisting research on the topic, it is not surprising that the criteria for inclusion in it were very broad. We were therefore interested in how many of these cases (hereafter referred to as “tool use”) would qualify as tooling, and whether the application of a more strictly defined criterion would reshape the outcome of past comparative research.

### 4.1 Methods

We systematically surveyed all tool use examples documented by Shumaker et al. (2011). Specifically, we scored each tool use example derived from Shumaker et al. (2011) as to whether it qualified as tooling, according to Fragaszy and Mangalam’s (2018) definition. Additionally, we extracted the species (or the genus or family if the species name was not provided), the tool use mode (for definitions, see Table 1.1 in Shumaker et al., 2011), and the sources. For the tooling examples, we further assigned the apparent type of empirical support (anecdotal, observational, or experimental), the number and type of spatial relations involved, and which examples involved manufacture or associative tool use. Tooling observations of only one individual were considered as anecdotes (with the exception of single case studies in an experimental context). If there were no more than two anecdotes by different authors, we classified the tooling type as “anecdotal,” otherwise we scored the tooling type as “observational” and/or “experimental.” Experimental support was considered any research in which variables were manipulated by the experimenters, for example when the animals were provided with candidate tools and/or tool use opportunities. Following Fragaszy and Mangalam (2018), types of spatial relations included frame of reference (allocentric or egocentric), temporal dynamics (static or dynamic), and temporal order (sequential or concurrent). We did not evaluate specificity (placement, orientation, and geometric alignment) as we did not have enough information from Shumaker et al. (2011) to score this consistently. Tool manufacture was defined as “any structural modification of an object or an existing tool so that the object serves, or serves more effectively, as a tool” (Shumaker et al., 2011, p. 11). Associative tool use was defined as the use of more than one tool “in any combination to achieve an outcome” (Shumaker et al., 2011, p. 19), which includes sequential tool use, composite tool use, metatool use, and secondary tool use. The data file as well as the R scripts of all analyses and visualizations can be found in the associated online repository at https://github.com/cvoelter/tooling_topiCS.

In order to correct for repetitive and redundant examples (i.e., multiple sources documenting the same type of tool use in a genus) in our analysis, we merged all tool use examples within a genus or taxonomic group in which similar objects (e.g., stick-like objects) were employed with a similar action for the same apparent purpose (e.g., raking an object within reach) and the same frame of reference. The apparent purpose was determined by the description provided by Shumaker et al. (2011). These merged examples are labeled as “submodes.” These submodes go beyond Shumaker et al.’s (2011, pp. 13–14) original 22 modes^2^, which serve as general categories of tool use behavior, whereas each of the submodes represents a unique type of behavior. For example, Shumaker et al.’s (2011) “reach” mode corresponded in our analysis to a number of different submodes depending on their apparent purpose, such as accessing another object (reach_access), assessing the depth of a pond (reach_assess), getting attention by someone else (reach_attention), or accessing a means for locomotion, such as a vine (reach_locomotion). Thus, we extracted the number of unique submodes to indicate the variability of tool use documented within a taxon. The mode and submode definitions and the data table are available as part of the Supporting Information. We reported the number of submodes only at the genus level (or at higher taxonomic levels) because the species was often unknown or had not been mentioned by Shumaker et al. (2011).

As a certain degree of subjectivity was involved in the assessments within the survey, the four co-authors who were involved with its creation independently checked the scorings of the other raters. In the few cases of disagreement by one of the raters, the cases were discussed and resolved among the group. In such cases, the default was toward the more conservative criteria (e.g., if it was unclear that a case involved associative tooling, it was judged negatively).

### 4.2 Results

Our survey yielded a total of 201 tool use submodes, 81 (40.3%) of which could be categorized as tooling (at least in some instances). Traditional tool use was identified in 221 genera; tooling in 76 genera. Across all tool using genera, 39.8% of all observed tool use submodes were categorized as tooling. Per tooling genus, there was an average of 3.7 tooling submodes (SD: 8.0; range: 1–53). Of all scored tooling submodes across genera, 55.4% were classified as anecdotal; 44.6% as observational and/or experimental; 19.4% were directed to the animals’ own body (egocentric), whereas 80.6% were directed at an external target (allocentric); 29.1% involved tool manufacture and 7.2% included associative tool use.

**Fig. 1:**
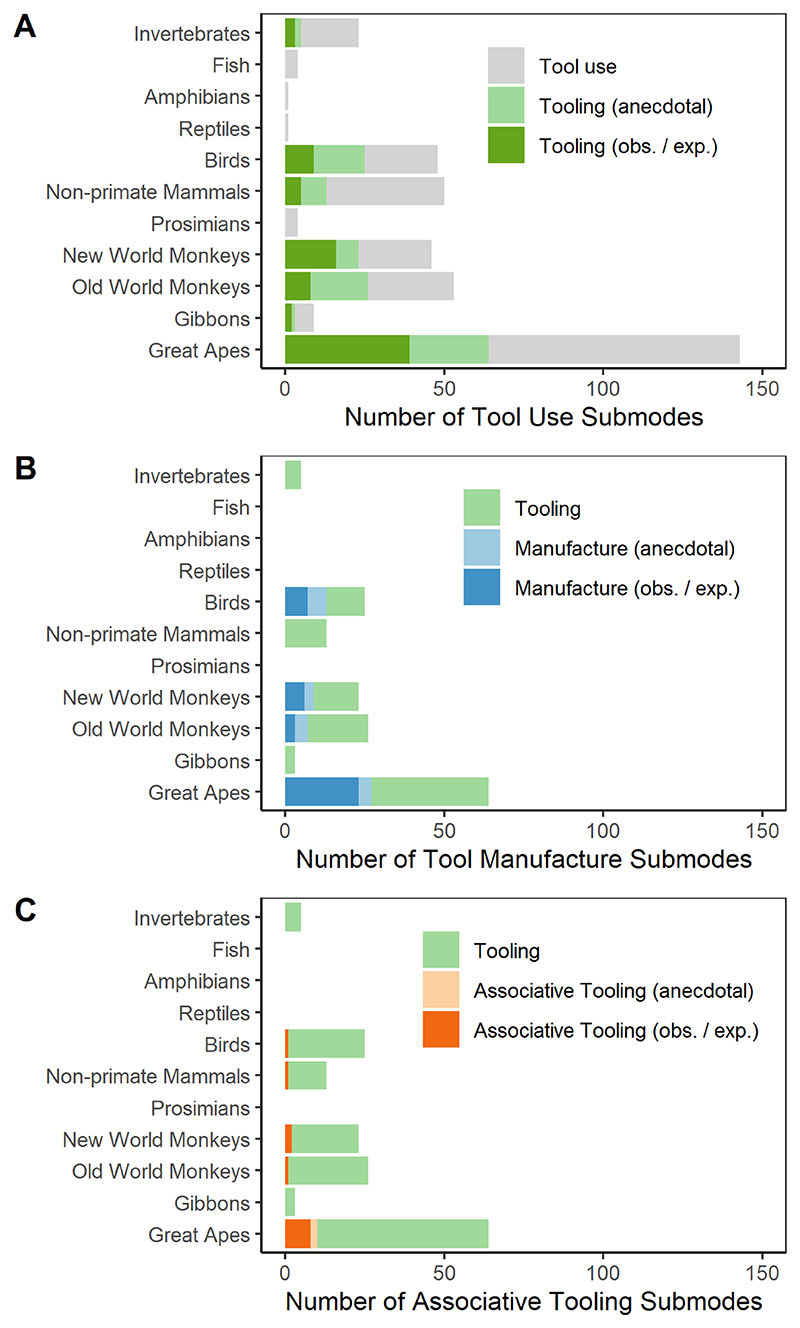
Bar plots showing the phylogenetic distribution of (A) the number of tooling submodes out of all tool use submodes documented by Shumaker et al. (2011), (B) the number of tool manufacture submodes out of all tooling submodes, and (C) the number of associative tooling submodes out of all tooling submodes. The light green bars in (B) and (C) represent all tooling submodes per group reported in Shumaker et al. (2011), including the anecdotal ones.

**Fig. 2:**
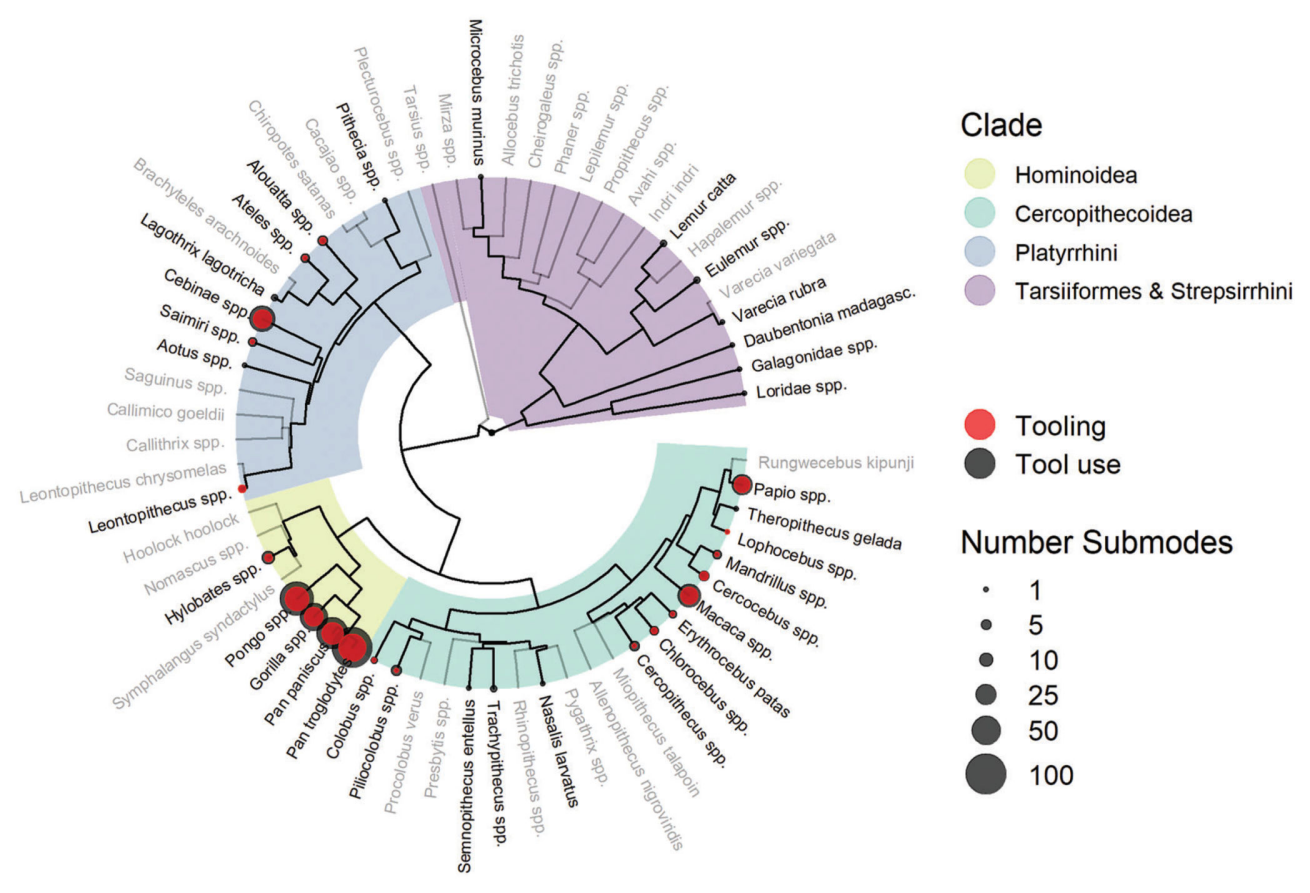
Cladogram of primate genera based on 10kTrees (Arnold, Matthews, & Nunn, 2010). For each genus, the black tip labels indicate the genera for which tool use examples have been reported in Shumaker et al. (2011). The size of the black and red points is proportional to the number of tool use and tooling submodes, respectively. We report the tool use data only at the genus level (except for the *Pan* genus, for which we show the data at the species level, and capuchin monkeys, galagos, and lorisids, for which we show the data at the subfamily level, *Cebinae* spp., or family level, *Galagonidae* spp. and *Loridae* spp.) due to many tool use cases reviewed by Shumaker et al. (2011) that did not provide the species name, but only the genus (or subfamily/family). To simplify the cladogram, we chose one species per genus to represent the genus, and dropped the other species from the cladogram.

The number of tooling submodes across different taxa is shown in Fig. 1A, which shows that (as of 2010) primates clearly provided the most tooling submode cases. Apart from primates, tooling was reported in nonprimate mammals, birds, and invertebrates. A large proportion of these tooling submode reports across the seven listed taxa were based on anecdotal evidence (48.4% overall), particularly the nonprimate cases (e.g., in birds 64.0% of the tooling submode reports were anecdotes^3^). Within the primate order, tooling was observed in multiple genera of apes (Hominoidea), Old World monkeys (Cercopithecoidea), and New World monkeys (Platyrrhini), but not in prosimians (Tarsiiformes and Strepsirrhini). Tooling appeared to be particularly abundant in the great apes (Hominidae; *P. troglodytes, P. paniscus, Pongo* spp., *Gorilla* spp.), capuchin monkeys (*Cebinae* spp.), macaques (*Macaca* spp.), and baboons (*Papio* spp.; Fig. 2). Within birds, tooling reports came from seven different families and nine different genera; 35.7% of the substantiated reports (i.e., excluding anecdotal evidence) were from the corvid family. Apart from the primates and birds, there were only a few tooling cases for which there was more than anecdotal evidence. Evidence (excluding anecdotes) from nonprimate mammals is limited to sea otters (*Enhydra lutris*), elephants (*Loxodonta africana*/unspecified), degus (*Octodon degus*), and pocket gophers (*Thomomys bottae*). Weaver ant (*Oecophylla, Polyrhachis, Dendromyrmex*) and digger wasp (*Ammophila, Sphex*) species, as well as one species of assassin bug (*Salyavata variegata*), provide the only nonanecdotal examples of tooling in invertebrates.

**Fig. 3:**
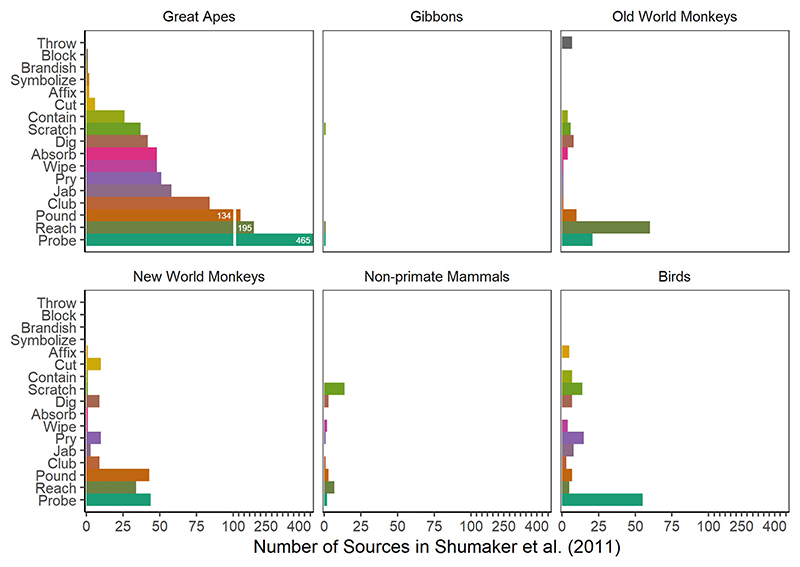
Distribution of the number of references per tooling mode across six different phylogenetic groups (based on Shumaker et al., 2011). The scaling of the *x*-axis changes from the value of 100 (by a factor of 0.15) to depict the large number of sources for the modes pound, reach, and probe in great apes (exact numbers printed on the bars). The gap in the corresponding bars indicates the change in the scale.

**Fig. 4:**
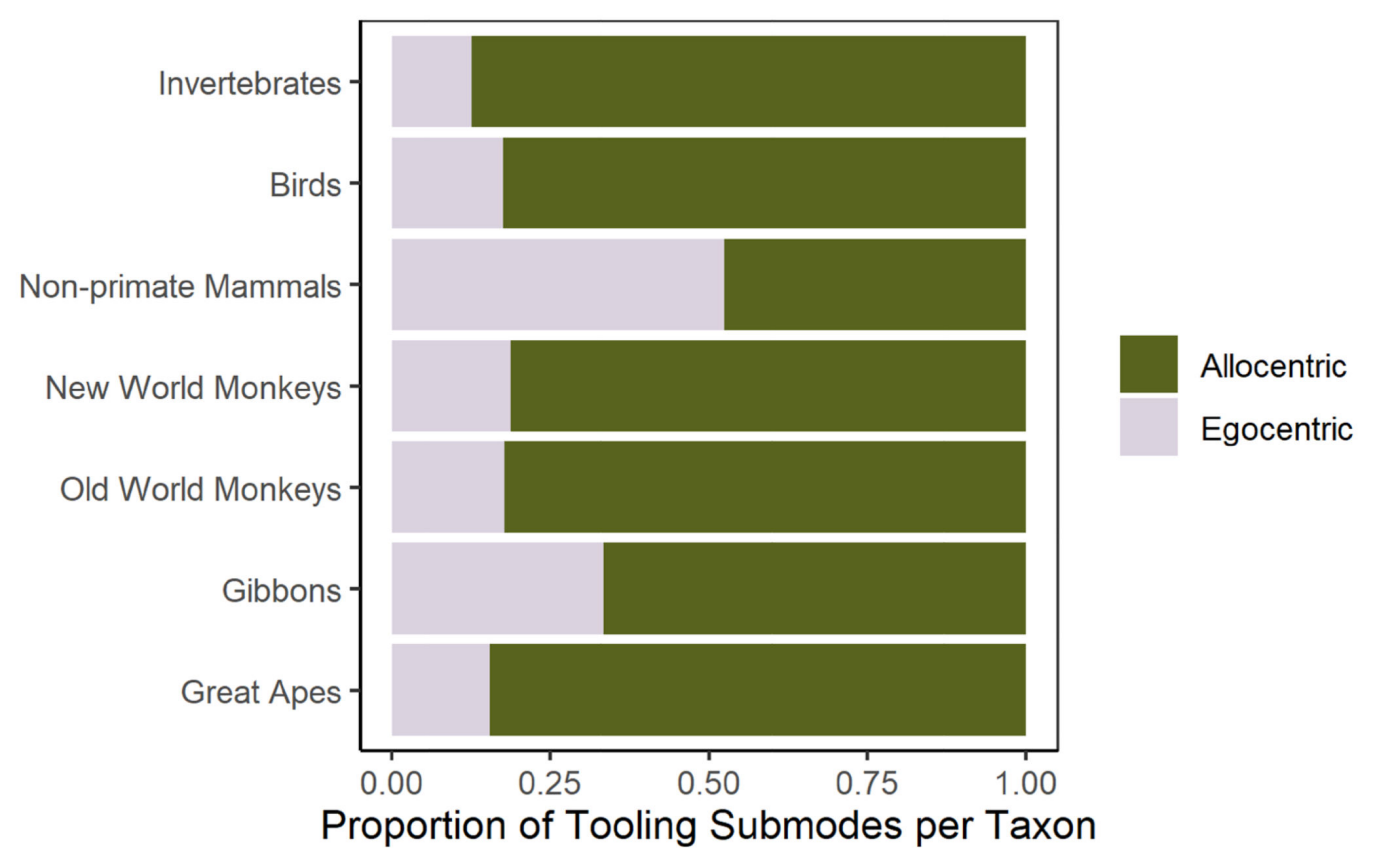
Bar plots showing the proportion of allocentric/egocentric tooling submodes within each taxonomic group based on the tool use examples documented by Shumaker et al. (2011).

Next, we divided the tooling examples by different tool use modes (according to Shumaker et al., 2011) to examine the number of citations given for the various taxa (Fig. 3). We found that probing was the most frequently referenced mode for the primates, followed by reaching and pounding. For birds, probing was by far the most commonly reported tooling mode. Among the few cases of tooling documented in invertebrates, the most commonly documented modes were affix (three genera of weaver ants) and pound (two genera of digger wasps). The most referenced tooling mode found in nonprimate mammals was scratching (of their own bodies). In fact, the largest proportion (52.4%) of egocentric submodes within genera occurred in nonprimate mammals, followed by 33.3% in gibbons, 18.8% New World monkeys, 17.8% Old World monkeys, 17.5% in birds, 15.5% in great apes, and 12.5% in invertebrates (see Fig. 4).

The most common apparent purpose according to the tooling submodes was “access” to some object or resource, at least for primates (great apes: 18.8%; gibbons: 66.7%; New World monkeys: 26.1%; Old World monkeys: 34.6% of all unique submodes per group) and birds (36.0%). For nonprimate mammals, the most common purpose of unique tooling submodes was “autogroom” (30.7%); for invertebrates, tooling was evenly distributed among “clamp,” “close,” “glue,” “hunt,” and “pack” (20% each).

Tooling submodes involving tool manufacture were reported for all of the great ape species, 18 species of birds (including five species of the family Corvidae), and multiple species of Old World monkeys and New World monkeys (see Fig. 1B); 33.3% of all tooling submodes involving tool manufacture relied exclusively on anecdotal evidence. Associative tooling cases were only reported for great apes (*Pongo* spp., *Gorilla* spp., *P. paniscus, P. troglodytes*), Old World monkeys (chacma baboons, *Papio ursinus*; rhesus macaques, *Macaca mulatta*; Japanese macaques, *Macaca fuscata*), New World monkeys (capuchin monkeys, *Cebinae* spp.), one bird species (New Caledonian crows), and one nonprimate mammal (sea otters, *E. lutris*; Fig. 1C); 10.0% of all associative tooling submodes were only based on anecdotal evidence.

### 4.3 Discussion

As expected, given the additional qualifiers of the tooling definition, tooling appears to be limited to a smaller number of species, with nearly two-thirds of genera considered to be tool users, but not considered to be toolers. Similarly, the majority of tool use behaviors did not qualify as tooling, which includes the majority of Shumaker et al. (2011) modes. Fragaszy and Mangalam (2018) argued that only 10 of these modes could be considered tooling, which we also found to be typically the case in our own analysis.^4^

Only a quarter of tooling types involved tool manufacture, and even less included the use of more than one tool or a composite tool (associative tool use). This is to be expected, as both tool manufacture and associative tool use often require a level of complexity beyond that required of simple tooling, such as inhibitory control or even planning (e.g., obtaining or making a tool to use later). Interestingly, while manufacture and associative tooling were relatively rare, their support was less often anecdotal compared to other tooling cases (Fig. 1B and C). This is perhaps not surprising as artificial laboratory setups are often necessary to elicit the more complex behaviors from animals that may be rare or more unlikely to arise in the wild (i.e., captivity bias; Haslam, 2013).

Although 80% of the tooling submodes consist of allocentric actions, remarkably the non-primate mammals, which had a limited number of tooling cases, had a greater proportion of egocentric submodes: self-grooming was their most common purpose for tooling. Fragaszy and Mangalam (2018) have hypothesized that egocentric relations are easier to manage than allocentric ones, in which case one might predict that this type of tooling should be more common and arise first in species with limited tooling abilities, as may be indicated here in the nonprimate mammals.

At first glance, this hypothesis appears to be contradicted by the data on invertebrates, which lies at the other extreme, with all well-supported evidence for tooling in insects being allocentric in nature. However, the data on insects indicate a different possibility; all of the few tooling cases are unusual in that a single type of tooling behavior is shared among related species. For instance, three genera of weaver ants, *Oecophylla, Polyrhachis*, and *Dendromyrmex*, are all reported to hold larvae that produce silk secretions against their nests in order to bind them together; similarly, two digger wasp genera, *Ammophila* and *Sphex*, both use objects to pound and pack soil around their burrows (Shumaker et al., 2011). The only other case involves one species of assassin bug (*S. variegata*), which holds termite corpses as lures for other termites (McMahan, 1982; McMahan, 1983); however, recent research has revealed that the feather-legged assassin bug (*Ptilocnemus lemur*) also uses a lure, though in this case it uses its own leg (Bulbert, Herberstein, & Cassis, 2014). These reports hint to the evolution of stereotyped, inflexible tooling, which can be identified by the use of “a single tool for a single purpose in a particular context” (Call, 2013, p. 5), typically in species showing only one type of tooling. How these evolved tooling specializations may fundamentally differ from flexible tooling is unspecified within the current tooling framework. We believe that the addition of flexibility criteria with respect to the number of different tooling modes, tool objects, purposes, and contexts (Call, 2013) in the context of tooling theory would be a particularly promising avenue for future theoretical and empirical work.

Although one indicator of potential inflexible tooling may be the presence of only one sub-mode in a taxon, one cannot make this assumption for every species showing limited forms of tooling, as this may also simply reflect a lack of research effort. Along these lines, while great apes have the highest diversity of submodes, the fact that they are also the most commonly researched animals must be taken into account. The number of sources per taxonomic group and tooling mode suggests that, at least until 2010, the great apes were clearly overrepresented in the tool use literature (see Fig. 3). This bias might be partly due to historic reasons (tool use research with great apes dates back to the studies by Köhler, 1917), elevated research attention due to their relatedness to humans, as well as an extraordinary diversity of tool use behaviors in the great apes compared to other taxa (see McGrew, 2013). A recent systematic review of comparative cognitive research with nonhuman primates published between 2014 and 2019 revealed that the great apes appeared in 38% of all surveyed studies, confirming that the great apes are still overrepresented among primates in contemporary research (ManyPrimates et al., 2019a).

Keeping this in mind, the tooling diversity within the primate order (see Fig. 2) stands out in a few families and genera, most notably the great apes, capuchin monkeys, macaques, and baboons. Tooling appears to be rare in the other New World monkey families and gibbons, and completely absent in prosimians. A more systematic investigation of tooling abilities as part of a large-scale collaborative effort, for example within the ManyPrimates et al. (2019a, 2019b) framework, might allow us in the future to conduct phylogenetic analyses and thereby reconstruct the evolutionary history of tooling within the primate order.

Compared to birds and nonprimate mammals, a larger proportion of the primate data are derived from observational and experimental studies rather than anecdotal data, which may also reflect greater research effort (see Fig. 1A). However, even though the proportion of anecdotal data is smaller for primates, the sheer number of anecdote-only supported submodes in the literature is astonishing. This indicates that there is a significant number of possible tooling submodes in many species awaiting confirmation by more systematic research efforts. Some of these anecdotes might also turn out to be false or unreliable. The latter possibility has been discussed extensively by Sándor and Miklósi (2020) who have advocated for reframing anecdotes as “behavioral rarities.” They argue that there are too many possible explanations for a rare behavior, including chance accidents, malfunctions, and developmental noise, for any causal interpretations or evolutionary inferences to be made from them. They also note that unusual behaviors may arise from unnatural environments such as captivity; certainly, we found a large number of one-time tooling behaviors accounted for by enculturated apes such as the bonobo Kanzi^5^ that are unlikely to ever occur in the wild. Sándor and Miklósi (2020) argue that anecdotes by their nature should not be considered evidence, and we agree that any interpretations of these behaviors should be made with an abundance of caution. A higher standard of evidence certainly should be required before we consider an animal a “tooling” species.

This is not to detract in any way from the value of anecdotes and single case studies, which may serve as indicators of a species’ potential abilities, opening up avenues for future research (Bates & Byrne, 2007). Two especially pertinent examples can be seen in the time span since Shumaker et al.’s book was published in 2011 and the present: the kea parrot and the Goffin’s cockatoo, also known as the Tanimbar corella. Shumaker et al.’s (2011) only reference to a Goffin’s cockatoo is Boswall’s (1983) brief secondhand account of a pet picking up a small container and using it to strike a hanging bell. Yet in 2012, it was fortuitously discovered that the Goffin’s cockatoo could spontaneously innovate and manufacture stick tools (Auersperg et al., 2012). Since then, a number of Goffin’s cockatoos have demonstrated tooling, exhibiting advanced cognitive abilities such as social transmission of tooling through emulation learning (Auersperg et al., 2014), making the same tool type from different materials (Auersperg, Borasinski, Laumer, & Kacelnik, 2016), innovatively bending and unbending hooked tools (Laumer, Bugnyar, Reber, & Auersperg, 2017), considering tool functionality for use and manufacture (Auersperg, Köck, O’Hara, & Huber, 2018; Beinhauer, Bugnyar, & Auersperg, 2019; Laumer et al., 2016), and safekeeping of tools for future use (Auersperg, Köck, Pledermann, O’Hara, & Huber, 2017). In a similarly striking case, the only mention of kea in Shumaker et al. (2011) is from a lay article in *Avicultural Magazine* from 1936, in which captive keas are described as scooping water with cups and tins in play (Porter, 1936). However, it has now been revealed that kea can engage in innovative tooling in the lab (Auersperg et al., 2011; Auersperg, Gajdon, & Huber, 2010; Auersperg, von Bayern, Gajdon, Huber, & Kacelnik, 2011; Lambert et al., 2017), and both kea and Goffin’s cockatoos may innovate tool use in the wild, but more research is required in both cases for a clear confirmation (Goodman, Hayward, & Hunt, 2018; Osuna-Mascaró & Auersperg, 2018).

This illustrates the limitations of focusing our analysis on Shumaker et al. (2011), in that research that occurred after 2010 is not accounted for. This is particularly apparent in the associative tooling and manufacture results for birds, which, for instance, would be quite altered by the recent research in Goffin’s cockatoos and kea. We were also reliant on Shumaker et al.’s (2011) reports and sources, as we did not perform a comprehensive survey of every reference ourselves, although we did consult a number directly when questions arose. Nevertheless, we believe that the current survey allows for identifying broad trends with respect to the phylogenetic distribution of tooling and the behavioral diversity of tooling within different clades.

## 5 Concluding remarks

Those aiming to understand ‘tool use’ behavior in diverse taxa from a biological perspective using conventional ethological definitions of the phenomenon are trying to build an enduring structure from a bag of confetti. (Fragaszy & Mangalam, 2018, p. 181)

In the light of the current replication crisis in the behavioral sciences, it is clear that tool use research needs higher standards of evidence before making extraordinary claims about a species, especially before this information is incorporated into broader theories and disseminated into the media as fact. As discussed, this is not to disparage the value of anecdotes, but a call for careful reporting and a caution against placing untoward weight on them, especially considering their high historical prevalence in the data.

Furthermore, in order to increase the replicability and interpretability of tool use research, a more cognitively coherent framework needs to be adopted. We believe that Fragaszy and Mangalam’s (2018) tooling concept fulfills this requirement. Moreover, it has strong neurocognitive underpinnings that support tooling as a distinct phenomenon from other types of tool use/object manipulations, particularly in the creation and management of a body-plus-object system. The tooling framework offers a solid foundation for making comparisons between species and generating hypotheses. We do not believe, however, that tooling should replace tool use as a concept, but rather that it is a more useful construct for the types of tool use behavior that fall under it, and a clarion call for similarly rigorous constructs for those phenomena that do not.

One of the main critiques of Fragaszy and Mangalam’s (2018) tooling theory was its neglect of the cognitive side of motor control and a call for a more integrative model (Osiurak & Danel, 2018). We hope that we have succeeded in beginning this work by contributing a neurocognitive perspective, and believe that there is a fruitful future for other elaborations of the tooling framework. To date, tool use research has been largely directionless and plagued by insufficient evidence; it is past time we stop tooling around.

## Supplementary Material

Supplementary Information

## Notes

1 “…a tool-using performance in an animal or bird is specified as the use of an external object as a functional extension of mouth or beak, hand or claw, in the attainment of an immediate goal. This goal may be related to the obtaining of food, care of the body, or repulsion of a predator, intruder, etc. If the object is used successfully, then the animal achieves a goal which, in a number of instances, would not have been possible without the aid of the tool.” (van Lawick-Goodall, 1971, pp. 195–196).

2 Shumaker et al.’s (2011) 22 modes include (1) absorb; (2) affix/apply/drape; (3) bait/entice; (4) block; (5) brandish/wave/shake; (6) club/beat; (7) contain; (8) cut; (9) dig; (10) drag/roll/kick/slap/push over; (11) drop; (12) hang; (13) insert and probe; (14) jab/stab/penetrate; (15) pound/hammer; (16) prop-and-climb/balance- and-climb/bridge/reposition; (17) pry/apply leverage; (18) reach; (19) scratch/rub; (20) symbolize; (21) throw; (22) wipe. See the Supporting Information for Shumaker’s (2011) definitions.

3 This appears to be largely due to the high volume of anecdotes found in birding and avicultural magazines.

4 Modes associated most often with tooling included club, cut, dig, jab, pound, probe, pry, reach, scratch, and wipe; predominantly nontooling modes included absorb, affix, bait, block, brandish, contain, drag, drop, hang, prop, symbolize, throw.

5 For example, in Shumaker, Walkup, and Beck (2011), there are reports of Kanzi extinguishing a candle flame with a knife tip, brushing a toy’s teeth with a tooth brush, and playing a xylophone with xylophone hammers.
